# 

**Published:** 1908-05-15

**Authors:** 


CONTENTS

Event and Comment - - - - - - - - - - 217

Causes of Failure in the Use and Application of Porcelain, *By Chalmers J. Lyons, D.D.S.* 220

Gold Inlays, - - *By Dr. Mortimer V. Hopkins, D.D.S.* 229

Mouth Breathing; its Cause and its Consequences, *By Woods Hutchinson, A.M., M.D.* 248

Indents - - - - - - - - - - - - 263

Announcements - - - - - - - - - - 268

